# The Social Relevance of Numbers: Insights from Animal Studies

**DOI:** 10.3390/life15111775

**Published:** 2025-11-19

**Authors:** Matteo Macchinizzi, Arianna Felisatti, Rosa Rugani

**Affiliations:** Department of General Psychology, University of Padua, Via Venezia, 8, 35131 Padova, Italy; matteo.macchinizzi@unipd.it

**Keywords:** numerical cognition, number sense, social intelligence hypothesis, social cognition

## Abstract

Number processing offers significant adaptive advantages, enabling animals to navigate their environment and maximize survival outcomes. Extensive evidence across species demonstrates that numerical cognition is a ubiquitous cognitive trait that enhances fitness by supporting critical behaviors such as foraging, hunting, and intergroup conflict. In this review, we examine the evolutionary and developmental roots of numerical cognition, focusing on its functional role in social contexts. First, we report research findings on the use of numerical information in various social behaviors among a wide range of animals. Then, we discuss how selective pressures arising from social complexity, like group size, hierarchical structure, and cooperation, contribute to the refinement of numerical abilities during social interactions. Finally, we explore how early social deprivation during ontogeny may shape the development of numerical cognition. We present a novel and integrative perspective on the evolutionary and developmental link between numerical skills and social life.

## 1. Numerical Cognition in Animals

Since the beginning of the 20th century, animal numerical competences have been vastly documented, and in the last decades, the interest in cross-species numerical cognition has constantly increased [1,2,3]. The first inquiry into numerical abilities in animals was quite unfortunate, marked by the famous case of Clever Hans. Hans was a horse that was trained by Wilhelm von Osten in the early 1900s to solve complex arithmetic calculations by tapping the results with his hoof. Initially believed by most of the scientific community, it was later revealed that Hans was not actually solving mathematical tasks through counting. Instead, it was a classic example of the Observer effect: the horse was detecting and interpreting unintentional behavioral cues from its experimenter. Hans would continue tapping until he detected these cues, which indicated when he had reached the correct answer [4].

In the 1930s, a systematic investigation of numerical abilities in animals started. The pioneer was Otto Koehler, who studied numerical cognition in several birds such as jackdaws (*Corvus monedula*), budgerigars (*Melopsittacus undulates*), and pigeons (*Columba livia*) [5]. A few years later, Jacky Emmerton and colleagues (1997; 1998) consolidated Koehler’s findings and documented pigeons’ ability to discriminate relatively smaller quantities (1,2,3) from larger ones (4,5,6,7) [6,7]. As research in this area progressed, evidence of numerical abilities emerged across diverse species, including rats (*Rattus norvegicus*) [8], macaques (*Macaca mulatta*) [9], and domestic chicks (*Gallus gallus*) [10].

Investigation of numerical abilities in animals and newborn humans has revealed the presence of a **Number sense**, a system for numerosity estimation [11,12]. This system emerges in the first days of human life. Being pre-symbolic and pre-verbal, Number sense has been proposed to be the foundation for more complex numerical reasoning and symbolic mathematical learning [13,14,15].

The phylogenetic ubiquity [16,17,18,19,20,21,22] and precocious availability [23,24,25,26,27,28] of the Number sense have led researchers to consider it as one of the core systems present at birth, which contribute to representing the most relevant environmental aspects such as objects, agents, spatial relationships, social partners, and numerosity [29,30,31]. Number sense involves two subsystems: the *Object File System* (OFS) and the *Analogue Magnitude System* (AMS). OFS supports the ability to rapidly and accurately process up to three/four elements by identifying each object based on its peculiar features (e.g., color, shape) and opening a file for each object in the working memory [32]. Instead, AMS enables estimations of numerosity beyond four as well as other magnitudes such as length, duration, and weight [33]. Remarkably, AMS follows Weber’s law [34,35], according to which the discriminability between two magnitudes becomes increasingly less accurate and slower as the ratio approaches 1: in fact, the ratio effect reflects a combination of the *numerical distance effect* [34,36,37] and the *numerical size effect* [38].

This fundamental cognitive ability is rooted in specialized neural circuits that have been increasingly mapped across species [12,39], with number processing engaging different brain regions [40]. Although the location and mechanisms of numerical skills are best understood in the human and primate brain, neurophysiological evidence in vertebrates, such as mammals, birds and fishes, has demonstrated that the *pallial regions* of the *endbrain* are involved in number representations [41,42]. The discovery of number neurons in the brains of different animal species has shed light on the neurobiological basis of the Number sense [43,44,45,46,47,48]. Intriguingly, these neurons are localized in homologous structures across species: the *nidopallium caudolaterale* in birds and the *prefrontal cortex* in primates [49].

This shared neurobiological foundation underlies the Number sense, and it encompasses various cognitive abilities [50], which serve as precursors for the subsequent development of higher numerical skills such as quantity and proto-numerical discrimination, proportional reasoning, and ordinal processing [51,52].

**Quantity discrimination** is the ability to differentiate between two sets of items to determine which is “more” or “less” numerous [31]. Quantity discrimination has been documented in different species and taxa, including primates [53,54,55], mammals [21,56,57], birds [58,59,60,61], fish [62,63,64,65,66], amphibians [67,68,69], reptiles [70,71,72,73], and even invertebrates [74,75,76,77,78,79].

Foraging behavior provides a classic context for observing quantitative discrimination. The seminal study by Krebs and colleagues (1974) demonstrated that various species preferentially select numerically advantageous food sources, consistent with the predictions of the Optimal Foraging Theory [80]. According to it, animals maximize energetic gain by choosing food patches that provide the greatest rewards relative to the time and energy expended [80]. This would lead animals to choose patches with more abundant food items. However, it is important to note that foraging could be influenced by non-numerical factors, such as food size. Therefore, animals may sometimes prioritize larger items over more numerous ones. Indeed, changes in numerosity (the number of items in a set) often correlate with changes in continuous variables like volume, surface area, density, and contour length [81]. To determine if animals truly perceive discrete numerosity, researchers carefully controlled continuous variables in laboratory settings. The discovery that animals are capable of discriminating between sets based solely on numerosity indicates **proto-numerical** rather than just quantity discrimination abilities [47,82,83,84,85].

Effective foraging in competitive environments critically depends on the ability to understand and use **proportional reasoning**. In a pivotal experiment, free-living mallards were offered different numbers of pieces of bread thrown at different rates and on opposite sides of a lake. Birds divided themselves between the resource patches as if they were simultaneously considering the amount of food and the number of animals feeding at each site [86]. The ability to use proportional reasoning is present across diverse taxa, including birds [87,88], fish [89], and primates [90,91].

In addition to foraging, numerical cognition also plays an important role in reproductive behaviors such as parental care and mate selection, thus shaping adaptive strategies across various species. For instance, male mosquitofish (*Gambusia holbrooki*) demonstrate a preference for groups with more females, likely to increase their mating opportunities [92]. In fish populations, males often adjust their reproductive tactics, such as sperm allocation, based on the number of males and females [93]. Similarly, male mealworm beetles (*Tenebrio molitor*) modulate the amount of time spent on mate guarding based on the number of rivals encountered. This strategy helps them to mitigate sperm competition risk and optimize reproductive success [94,95]. Numerical abilities can also enhance offspring survival. For example, American coots (*Fulica americana*) reduce brood parasitism by keeping track of the number of eggs they lay and rejecting extra parasitic individuals [96]. These examples illustrate how numerical assessment adjusts behaviors throughout the reproductive process: from mate search and competition with rivals to offspring care.

This capacity to track items and process numerical information may have evolved particularly in social species such as primates, cetaceans, corvids, and parrots [59]. Indeed, within social environments, this capacity is likely to provide a key adaptive advantage during competition between groups and predation, as will be explored in the section *Social Dynamics Requires Numbers*.

Another fundamental aspect of number processing is **ordinality**. It encompasses two distinct cognitive abilities: (a) the processing of an item’s position within a series, and (b) the understanding of relative magnitude, where adding an item to a set makes it larger than the previous set but smaller than the following one [31].

The processing of an item’s position within a series has been demonstrated in various animal species, including rats [22,97], domestic chicks [98,99], nutcrackers [100], zebrafish [101], and honeybees [102]. Such ability enables animals to better orient themselves and navigate their environments, counting landmarks as reference points [102]. Instead, the understanding of relative magnitude has so far been documented in macaques [16], and pigeons [103].

These findings suggest that non-human animals can process ordinal relationships abstractly and infer hierarchical numerical rules. The ability to organize stimuli according to their numerical value, creating a sequential and linear arrangement where each element precedes the one that follows, is a key cognitive skill underlying the process of *transitive inference* [104]. Transitive inference is a deductive process through which individuals infer a relationship between two non-adjacent items based on their known relationships with a third item (e.g., if A > B and B > C, then A > C). This process relies on an internal representation of the entire sequence that shares mechanisms with the number sense, as evidenced by Weber’s law [16,105].

Relating objects or individuals is a fundamental building block for establishing hierarchical relationships within a social group [106]. Indeed, enumerating conspecifics and tracking dominance relations between individuals relies on core numerical mechanisms. These mechanisms offer clear adaptive advantages, including foraging, navigation, predator avoidance, hunting, and mating [107,108] (see Table 1). Are these numerical abilities merely useful for individual fitness or specifically shaped and required by the complexities of social living?

## 2. Social Dynamics Requires Numbers

**Figure 1:**
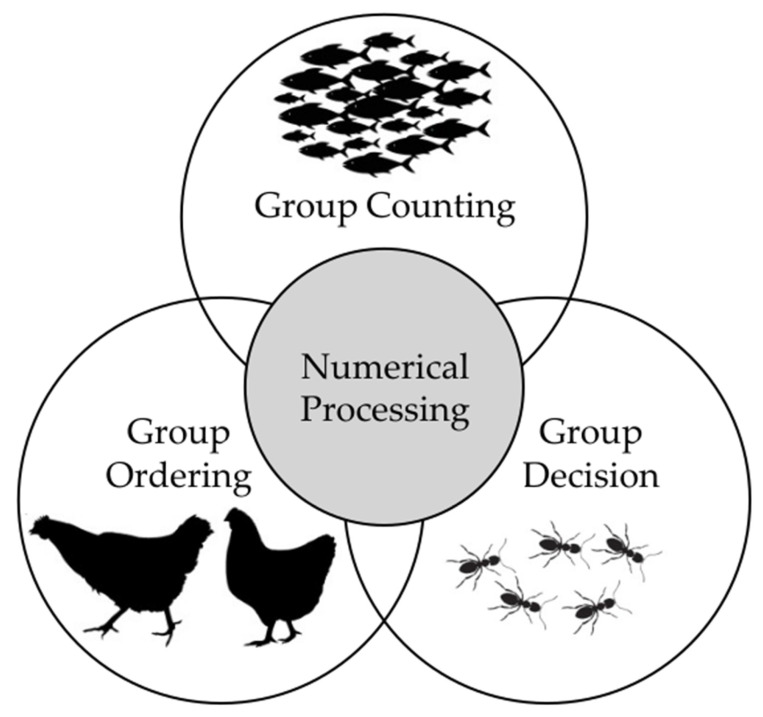
Numerical Processing in Social Contexts. Numerical information provides advantages during social behaviors such as inter-group conflict (assessing group size), navigating social hierarchy (ordering group members), and initiating collective action (e.g., estimating proportions of individuals involved in a specific behavior).

Within a broad adaptive landscape, social life presents a particularly complex set of challenges that leverage and may have driven the refinement of numerical processing. Numerical cognition thus plays a key role in group dynamics and constitutes a foundation for ecological success in many social species. Here, three key numerical processes used in social dynamics will be examined (Figure 1). First, the capacity to estimate the number of conspecifics in a group: a pivotal component in assessing competitive advantage, optimizing hunting strategy, and minimizing predation risk. Second, representing social hierarchies: another essential component in social life, helpful for minimizing aggressive encounters, injuries, or death. Finally, collective decision-making: a process that often relies on a numerical threshold, enabling efficient and cohesive group choices. Numerical processing advantages and their possible evolutionary pressure in social contexts are summarized in Table 2.

### 2.1. Counting the Group Members

A fundamental ability observed in most social species is the capacity to estimate the number of conspecifics in a group. For group-living species, the adaptive tuning of group size is critical, as it often affects fitness outcomes [135,136,137]. Social species frequently rely on proto-numerical discrimination to assess competitive advantage during intergroup conflicts, determining whether a rival group is larger or smaller, and playing a pivotal role in territory defense [109,138]. For example, in the seminal study of McComb and colleagues (1994) [109], female lions (*Panthera leo*) were less likely to engage in approach behavior when confronted with the simulated roars of three unknown intruders compared to a single intruder. The lionesses’ behavior, either alone or in a group, was strongly influenced by the ratio of defenders to intruders, underscoring the importance of numerical odds in social conflict. Similar numerical assessment during intergroup conflict was reported in wild spotted hyenas (*Crocuta crocuta*) [110], chimpanzees (*Pan troglodytes*) [111], black howler monkey (*Alouatta pigra*) [112], subdesert mesite (*Monias benschi*) [113] and in free-ranging dogs (*Canis lupus familiaris*) [114].

This numerical skill is also particularly crucial for social carnivorans in making hunting decisions. Proto-numerical assessment allows them to select foraging patches with greater prey availability or less dangerous prey and coordinate cooperative hunting efforts [108]. For instance, in wolves, numerical assessment helps balance the risks and benefits of attacking large and dangerous prey. Hunting a bison entails a much higher risk of injury than pursuing an elk, and numerical evaluation allows wolves to estimate whether the group size is sufficient for a successful hunt (usually packs of 9–13 wolves for bison, while 2–6 for elk) [108]. Numerical discrimination informs decisions on which foraging patches to exploit or which prey to target based on size, choices critically dependent on the size of the hunting party itself [139]. This widespread behavior aligns with Game Theory predictions, as numerical assessments are often prioritized over other factors during decision-making, maximizing the likelihood of success [138,140].

Crucially, estimating group numbers confers advantages not only for predators in hunting contexts but also to prey in defensive ones. The formation of social groups is a common evolutionary response to predation risk, providing individuals with several adaptive benefits [141]. The mechanisms that enhance safety of individuals in groups include the *confusion effect*, which makes it harder for a predator to single out and attack an individual prey animal; the *many-eyes effect*, which increases the likelihood of detecting predators; and the *dilution effect*, wherein an individual’s risk of predation decreases as the number of group members increases [142,143,144].

Many fish utilize their ability to choose larger, safer social groups when navigating unfamiliar or potentially dangerous environments [107,145]. For example, both guppies (*Poecilia reticulata*) can discriminate between 4 and 5 individuals [116] while three-spined stickleback (*Gasterosteus aculeatus*) can distinguish between 6 and 7 [117]. Moreover, mosquitofish show discrimination dependent on the ratio between the two sets: successfully discriminating 4 vs. 8 and 4 vs. 10, but not 4 vs. 6 or 4 vs. 7 [62]. This proto-numerical ability enables prey species to reduce their predation risk by preferentially joining larger groups.

The same capability of domestic chicks to spontaneously approach the larger group of conspecifics [133,146,147] has also been used to test **proto-arithmetic abilities**, namely the capacity to operate on non-symbolic numerical representations through basic addition and subtraction [31]. For example, in one study, newborn chicks reared with five objects (social companions) were able to track and compare group sizes when the objects disappeared one by one behind screens (e.g., 1 + 1 vs. 1 + 1 + 1). This ability persisted even under more complex conditions involving both addition and subtraction (e.g., 1 + 2 vs. 4 − 2), suggesting a spontaneous capacity to form and manipulate mental representations of quantity [133].

The findings reported in this first subsection highlight how social dynamics benefit from numerical abilities. Across taxa, competition and predation pressures have acted as strong selective forces shaping the evolution of proto-numerical discrimination. This skill emerged as a tool for survival, tightly linked to the social behavior of the species.

### 2.2. Ordering the Group

Alongside counting potential rivals, predators, or prey, animals living in social groups also navigate complex social systems. They must recognize individuals, track social relationships, and assess their status within the hierarchical order of the group. The ability to recognize the status of a conspecific is crucial to minimizing agonistic and aggressive encounters. Indeed, higher-ranking conspecifics typically have greater physical threats, and recognizing them is essential for avoiding direct confrontations that could result in injury or even death [148].

Using playback experiments, Bergman and colleagues (2003) [106] demonstrated that baboons not only recognize the rank orders of various group members but also understand which matrilineal kin group each individual belongs to. This indicates that primates organize social concepts hierarchically and highlights their sophisticated cognitive abilities. Hierarchy is not limited to primates. Ravens (*Corvus corax*), for instance, provide compelling evidence of hierarchical understanding [120]. Remarkably, ravens exhibit such responses not only for members of their own group but also for those of adjacent groups [148,149,150]. Similar findings have been observed in geese (*Anser anser*) [121], and domestic chickens (*Gallus gallus*) [122,123], suggesting that this ability is a fundamental cognitive building block in species with dominance hierarchies [151,152].

Another example comes from the study by Daisley and colleagues (2021) [122] in which transitive inference reasoning was studied in relation to chicks’ social rank and sex. The results showed that females outperformed males in this task. Moreover, females’ performance decreased linearly from the lowest to highest ranks, while in males, middle-ranked individuals had the best performance. These findings have been attributed to sex-specific social behaviors: female chickens typically form a rigid linear hierarchy, where low-ranking individuals closely monitor dominance relationships to avoid conflict. On the other hand, a few males control territories, with males of intermediate rank competing more for mating opportunities [153].

Crucially, invertebrates also exhibit transitive inference. Paper wasps (*Polistes dominula* and *Polistes metricus*), despite miniature brains, could organize color pairs into linear hierarchies and infer novel relationships. Notably, both species performed equally well despite *P. dominula*’s living in more complex social groups compared to *P. metricus*’ smaller groups [124]. This shared ability despite differing social complexity suggests that the cognitive processing of dominance hierarchies may be evolutionarily conserved, potentially linked to ancestral dominance behaviors rather than driven by current socio-ecological demands [124]. This contrasts sharply with honeybees (*Apis mellifera*), which fail transitive inference tests, likely reflecting the lack of ecological relevance of this cognitive ability in their non-hierarchical societies where social roles are fixed [154].

In conclusion, the ability to infer and predict social relationships is crucial for navigating the complex dynamics of group life, increasing the chances of survival by optimizing conflict resolution and reproductive success.

### 2.3. Group Decision

Group-living animals, beyond static assessment of companions, or the ranking order, rely on numbers for crucial dynamic and coordinated actions. Numerical processing has a key role in group decision making, which often relies on collective mechanisms. Among these, *quorum sensing* is a key process by which behavioral responses are triggered once a threshold number of individuals perform a key action [127]. To achieve this, individuals must correctly perceive and estimate the number of conspecifics engaged in the specific action. Crucially, such group-level responses often rely on relative proportions rather than absolute counts, consistent with Weber’s Law, which states that sensitivity to differences between numerical sets depends on their ratio rather than absolute difference [127].

Compelling examples come from white-faced capuchin monkeys (*Cebus capucinus*), where adult individuals use specific vocalizations (trills) to initiate or redirect group movement. The group typically responds and moves in the direction indicated by the trilling individual only after a sufficient number of group members appear to support the initiative, either by position or vocal reply [128]. Similar evidence from wild olive baboons (*Papio anubis*) revealed that collective movement is governed by shared, majority-based decision rules [129]. The likelihood of a baboon joining a group increases in an S-shaped curve as one subgroup gains a numerical advantage over another. However, baboons are only willing to compromise and follow a different direction when there’s minimal disagreement between subgroups about which way to go.

Similar mechanisms for group decision are present in other highly social species, such as African wild dogs (*Lycaon pictus*) [130], and meerkats (*Suricata suricatta*) [131]. This process enables individuals to choose among mutually exclusive alternatives while maintaining group cohesion [125].

In social insects, quorum decisions are vital for processes such as selecting a new nest site. For instance, in the Japanese ant (*Myrmecina nipponica*), the quorum thresholds for nest selection increase with colony size [125]. Ants employ an analogue magnitude mechanism to assess group size, so their accuracy diminishes with increasing stimulus magnitude due to limitations consistent with Weber’s Law [125]. This ratio-dependent mechanism implies that ants’ numerical discrimination abilities decrease as the size of the group grows, affecting their capacity for optimal collective actions like resource allocation during colony fission or coordinating foraging efforts.

Honeybees (*Apis mellifera*) also demonstrate quorum sensing in their decision-making processes. For example, they initiate swarming when 10–15 scout bees gather at a single location [132]. During house-hunting, both bees and ants (*Temnothorax rugatulus*) rely on quorum processes to select among potential nest sites [126,132].

The studies of quorum sensing in various species highlight the benefits of numerical competence in collective decision making. Numerical discrimination and ordinal processing are critical components of animal social behavior, shaping collective and individual behaviors across a wide array of species.

## 3. Evolutionary Pressure

The diverse scenarios outlined above highlight how numerical abilities contribute directly to social behaviors. Indeed, environmental and social demands act as potent evolutionary pressures in shaping cognitive traits. Numerical cognition may have emerged repeatedly across evolution, suggesting possible evolutionary convergence in phylogenetically distant taxa, highlighting its adaptive value [155]. In the present section, we expand this perspective by examining both ecological and social pressures that drive the evolution of cognition across species.

### 3.1. The Adaptive Value of Cognition

One of the most prominent hypotheses about selective pressure for cognitive development is the *Ecological Intelligence Hypothesis* (EIH) [156,157]. According to it, the drivers of cognitive evolution are ecological challenges, such as unpredictability of resource availability, habitat complexity, and predation risks [158,159,160,161,162]. Challenges such as parasitism [163], predation [164,165], habitat complexity [166], and foraging behavior [167,168] may have been selective pressures for variation in cognitive abilities, such as numerical processing. For example, in the case of extractive foraging aimed at obtaining inaccessible, hidden or protected foods, primates may have evolved higher cognition and intelligent tool use [157,169]. Likewise, the seasonal nature of many primate diets, such as frugivores, requires cognitive mapping, which is a representative expression of an individual’s knowledge about the spatial and environmental relations of geographic space [170]. Indeed, according to the EIH, memorizing the spatial and temporal availability of food could have been one of the pressures for the development of higher cognitive skills [170,171]. Analogous to observations in primates, compelling evidence exists for similar cognitive adaptations in birds. For example, scrub jays (*Aphelocoma californica*) and Clark’s nutcrackers (*Nucifraga columbiana*) hide food to ensure survival during periods of food scarcity, demonstrating a complex capacity for memory and planning [172,173].

EIH suggests that ecological challenges were the drivers of larger relative brain size, since activities such as food acquisition require spatiotemporal skills and/or manipulative skills, as mainly documented in primates [174,175,176,177]. Notably, corvids, compared to other avian groups, possess larger relative brain sizes and are known for their use of extractive foraging techniques that resemble primates’ techniques, such as the use of sticks to catch insects hiding in crevices [178].

Ecological factors, such as specialized diets, may directly select for numerical cognition. Black-handed spider monkeys (*Ateles geoffroyi*) are highly frugivorous primates and they need to optimize foraging in patchy, due to the ephemeral fruit distributions. To do so, they likely rely on quantitative assessments of food patches [179]. Indeed, they excel in complex food ratios discrimination (e.g., 4 vs. 5 items), aligns with the EIH predictions.

Nevertheless, spider monkeys also share a key social trait with great apes: they live in fission–fusion society, making it difficult to disentangle ecological pressures with social complexity. Their proficiency in abstract numerosity tasks (e.g., discrimination of dot arrays based on their numerosity when continuous variables are controlled) could also support the adaptive role of numerical cognition in tracking fluctuating subgroup composition.

### 3.2. The Social Intelligence Hypothesis

Along with ecological challenges, social living is also a critical driver for cognitive traits. This is the central argument of the *Social Intelligence Hypothesis* (SIH) [180,181,182], which states that enhanced cognitive abilities emerge in group-living species, because social living imposes selective pressures that demand advanced cognition [120,183]. As shown in the previous sections, numerical cognition plays a crucial role in social dynamics. It allows individuals to estimate and monitor group size, track social companions, navigate dominance hierarchies, and optimize competitive advantages during conflict. Numerical abilities may have been subjected to the same selective pressures proposed by SIH, thus supporting complex social behaviors and helping individuals to adapt to the challenges of social life.

Evidence from several taxa, including birds, fish, rodents, and primates, suggests that individuals who live in larger social groups exhibit better cognitive performance [184]. Existing literature illustrates how social factors can be the cause of intraspecific variation in cognitive performances, highlighting their relevance in influencing cognitive development, which in turn reflects on fitness [184]. Therefore, evidence suggests that an individual’s cognitive development is influenced by their social surroundings.

This relationship becomes evident also on the neural level: A large body of research uses measures of brain size or brain regions (e.g., neocortex, amygdala, cerebrum, cerebellum, and telencephalon) as a measurement for cognitive abilities [184,185]. For example, the *encephalization quotient* (a measure of brain size relative to body mass) varies significantly among species [186] and has been associated with variation in cognitive performance [187]. This is also true for the *neocortex*. The neocortex is a complex brain structure that controls and regulates higher functions such as spatial reasoning, planning and decision-making. Studies from numerous taxa (primates, bats, carnivorous mammals and cetaceans) found that neocortex size is positively correlated with social group size [188,189,190]. Such a correlation is supposed to stem from the need to remember, track and manage relationships in larger groups [191].

However, other studies seem to contradict the positive correlation between brain and group size [192]. Indeed, the link between sociality and brain size strongly reported in primates is not as clear in other groups, such as ungulates [193], lemurs [194], birds [195,196,197], corvids [198]. The use of neuroanatomical measures as a proxy for cognition, is still a topic of debate given both the negative [199] as well as positive findings [200], and also the fact that different taxonomic groups show similar cognitive abilities despite very different brain structures [201].

Diverging from the use of brain size as a proxy for cognition, the study of MacLean and colleagues (2013) focused on the association between group size and cognition across primates [202]. Their findings indicate that group size was a predictor of social cognition, measured as the ability to understand a competitor’s visual perspective. Instead, group size did not predict a non-social cognitive measure, i.e., inhibitory control in a food retrieval task. Another question that needs to be addressed when considering the SIH is which cognitive traits are expected to respond to evolutionary changes in social systems. To date, we can identify two hypotheses, one in line with the domain-specific hypothesis [203] and the other with the domain-general hypothesis [204]. According to the domain-specific hypothesis, greater social complexity leads to selection for cognitive skills that are specific to social life [205]. Contrarily, based on the domain-general hypothesis, social complexity favors both social and non-social cognitive skills [206]. However, it is important to point out that the relationship between group size and cognition is neither universal nor unidirectional. A recent study tested spontaneous proto-numerical discrimination in wild Western Australian magpies (*Gymnorhina tibicen dorsalis*). The results showed that individuals from smaller groups exhibited enhanced discrimination accuracy compared to those from larger groups [61]. Two primary explanations account for these findings: (a) smaller groups face higher intergroup conflict costs, selecting for better numerical assessment skills; (b) fewer group members increase vigilance demands (“many-eyes effects”), enhancing foraging-related discrimination [61]. This highlights how group size, along with task-specific demands and ecological context, can influence cognitive abilities in different ways, necessitating nuanced interpretations of cognitive adaptations.

### 3.3. Refining the Proxies of Sociality

The relationship between group size and cognitive abilities is highly debated, and recent research suggests that group size alone may not be an ideal proxy for social complexity [207]. A more nuanced approach is necessary, considering the type and quality of relationships among individuals [208,209,210]. Freeberg and colleagues (2012) proposed a definition of complex social systems as “those in which individuals frequently interact in many different contexts with many different individuals, and often repeatedly interact with many of the same individuals over time” [211]. This definition encompasses factors beyond mere group size, including mating systems, dominance styles, and breeding strategies [207]. Studies on primates and corvids living in fission–fusion societies (i.e., complex societies where there is frequent splitting and merging in subgroups of variable composition) have shown superior performance in cognitive tests assessing impulse control and behavioral flexibility compared to those in more stable groups [212,213]. However, these findings require careful interpretation. The cognitive demands of fission–fusion societies might indeed be different, but not necessarily greater, than those of more stable groups. For instance, smaller and more stable groups might develop different cognitive abilities due to higher survival pressures or the need for more intense cooperation among fewer individuals.

The ‘Machiavellian intelligence’ or ‘social brain’ hypothesis proposes that large brains are an adaptation to social complexity in long-term social groups [171,203]. Larger brains have also been found in groups where individuals need to make strategic decisions to manage conflicts, such as leading animals towards or away from places, deflecting aggression onto innocent third parties, or hiding the food in the hand or under the body [171,203,214]. Actually, it has been suggested that the most challenging societies are those where individuals live in multi-generational units, members are able to recognize each other, there is cooperation and/or competition for resources and the majority of learning happens during social development [215]. Social tactics that seem sophisticated, such as deception and cooperation, may be built on quite simple cognitive mechanisms: the main capacity that is needed is efficient memory, to differentiate individuals and keep track of the kinship networks, dominance hierarchies and mutual exchanges that constitute social life [216].

Even though results in numerous species, such as spotted hyenas [217], are very promising and once again sustain the SIH, at the moment, it is still necessary to further compare cognitive abilities among more and less complex social systems to affirm that the evolution of the brain and behaviors has occurred in response to social complexity. For example, from the Reader and Laland’s (2002) comparative analysis of 445 observations of social learning [218], no correlation was found between social group size and social learning frequency. Currently, there is still little evidence regarding social learning being an adaptive specialization to particular environmental demands, beyond favoring behavioral flexibility in general [219]. 

On the other hand, the evolution towards larger brains could also have ecological explanations. Two prominent hypotheses in this domain are the *Extractive Foraging Theory* [157] and the *Cognitive Mapping Hypothesis* [156]. The former posits that the cognitive demands of accessing limited embedded foods through tool use and complex object manipulation drove brain expansion in primate ancestors. The latter proposes that the challenge of locating scattered food sources in rainforests led to enhanced spatial memory, mental mapping, and enlarged brains. These perspectives show how the discourse on the driving forces for brain evolution is still open to debate [220]. Overall, Reader and Laland study [218] support the idea that the EIH and SIH should not be considered alternatives to one another, since the sources that favored the evolution of a large primate executive brain were multiple [171,188,220].

A recent meta-analysis of 103 studies across 17 taxonomic orders shows a correlation between sociality and cognition in various species, contexts, and measures, thus supporting the SIH as an evolutionary and developmental explanation for cognitive variations [185]. However, this analysis also revealed significant gaps in our understanding, particularly in underrepresented taxa, and called for more standardized cognitive and neuroanatomical tests.

Whether sustaining the SIH or the EIH, the driver of cognitive evolution could be considered the same in both cases, that is, individuals gathering and processing information to mitigate uncertainty [221,222]. Indeed, treating social and ecological variables as independent may not be the most appropriate approach. Social species often need to solve ecological problems in a social context, and social complexity itself may have evolved as a response to ecological factors [184,223]. This interplay between social and ecological pressures could lead to diverse cognitive adaptations that are not captured by simple measures of group size or social complexity.

Furthermore, while evolutionary pressures are significant, it is equally important to consider how sociality shapes cognitive development at the individual level. This raises questions about how insufficient or inappropriate social inputs affect brain development and cognitive function.

Future research should aim to disentangle the effects of different aspects of sociality, consider the interplay between social and ecological factors, and explore how these influences manifest across different species and ecological niches.

## 4. Effects of Early Social Deprivation

Early social experiences play a crucial role in shaping various aspects of cognitive development, including numerical cognition. Despite the effects of early social experience having been extensively studied in other cognitive domains [224], there remains a significant gap in understanding its specific role in the ontogenetic development of numerical cognition [107]. This section will explore the current understanding of how early social experiences influence the development of numerical cognition in animals.

To understand why early social experiences are so impactful, it is necessary to understand and define the concepts of *sensitive* and *critical periods*. *Sensitive periods* are a limited time window during development where there is a heightened potential for neural (re)organization in response to environmental inputs, whereas *critical periods* are a special class of sensitive periods where the neural system requires definitive inputs to organize and develop physiologically [225,226]. A well-known example of a sensitive period is song learning in songbirds, where young birds must be exposed to adult songs during a specific developmental window to learn their species-specific song patterns effectively. The timing of this sensitive period varies between species and can be influenced by both experience and hormones [227]. Instead, an example of a critical period is filial imprinting in ducks and domestic chickens, where within a few days of hatching, these animals imprint on stimuli that they identify as the parent [228,229,230]. It is noteworthy that the phenomenon of filial imprinting has been extensively utilized in domestic chicks as a paradigm for investigating numerical cognition [133,146,231].

While research directly linking early social experiences to numerical cognition is limited, some studies provide insights into this relationship. For instance, Bisazza and colleagues (2010) investigated the impact of social experience on numerical abilities in guppies (*Poecilia reticulata*) [115]. They found that at 20 days of age, guppies required social experience to discriminate large numerosities (4 vs. 8), whereas by 40 days, this ability emerged regardless of rearing conditions. This suggests that while maturation drives basic numerical capacities, social experience may speed up the refinement of number discrimination.

Recent findings in newborn chicks further support the idea that social context can enhance numerical discrimination. When reared with individually distinctive objects (treated as social companions), chicks successfully discriminated between sets of 3 vs. 4 items, a comparison they typically fail when the objects are all the same [134]. This improvement is thought to result from enhanced object individuation, which facilitates encoding in working memory via the Object File System. These findings suggest that social relevance and perceptual individuation can enhance non-symbolic numerical abilities by engaging working memory mechanisms dedicated to tracking individual objects [134].

The study by Sheardown and colleagues (2022) on zebrafish (*Danio rerio*) provides additional insights into the ontogeny of numerical cognition [119]. While this study did not manipulate social experience directly, it demonstrated that fish’s ability in proto-numerical discrimination improves with age (21–33 days post-fertilization). This raises questions about how social experiences might interact with maturation to shape numerical abilities.

Remarkably, social deprivation impairs various cognitive abilities [225,232], including working memory and attention, which are fundamental to numerical competence.

Working memory allows individuals to temporarily store and manipulate numerical information. For instance, when comparing two sets of objects, an animal must hold representations of both sets in mind simultaneously [133].

Attention is another key cognitive process supporting numerical competence. Indeed, the ability to selectively focus on relevant numerical information while ignoring distractors is crucial for numerical judgments. Social deprivation has been shown to affect attentional processes [233], which could consequently impact numerical tasks [234].

Some researchers hypothesize that the lack of social complexity in an early environment may result in a neural structure designed to deal with low complexity environments [235]. This evidence would be explained by the phenomenon of *synaptic pruning*, a process in which unused or less active neural connections are eliminated. Synaptic pruning is a central force in the remodeling of the brain’s neuronal connections throughout development in response to experience [236]. For example, animals raised in a deprived environment, such as in an empty cage with no conspecifics, toys, and novel stimuli, exhibit reductions in the number of synapses per neuron [237], the density of cortical dendritic spines [238], the branching and length of dendrites [239,240], and cortical depth [241]. While the direct link to numerical cognition is not established, such changes in neural connections could potentially influence the development of cognitive abilities, including numerical skills.

Critically, early social isolation disrupts oligodendrocyte maturation and myelination in the prefrontal cortex through specific molecular pathways [242] and induce long-term alterations of large-scale gene expression patterns in the brain [243,244].

For example, one study by Weaver and colleagues (2006) found that rats that received high levels of maternal care during the first week of life had increased expression of the glucocorticoid receptor gene in the hippocampus, compared to rats that received low levels of maternal care [244]. The glucocorticoid receptor gene is involved in the regulation of the hypothalamic–pituitary–adrenal axis, which is responsible for the body’s stress response, making those who received high levels of maternal care more resilient to stress later in life.

Similarly, in rhesus monkeys, early postnatal deprivation of maternal care induces differences in gene expression in the amygdala. Sabatini and colleagues (2007) identified GUCY1A3 as a gene whose expression was significantly reduced in monkeys separated from their mothers at one week of age [243]. GUCY1A3 is involved in the production of the enzyme guanylate cyclase, which plays a role in various processes, including synaptic plasticity, learning, memory, and stress response.

Moreover, as a result of a lack of complexity and the absence of sensory, cognitive, and social stimulation, there are other mechanisms affected, such as epigenetic regulation [245]. Early social environments can lead to persistent alterations of brain gene expression [246,247]. For example, juvenile cooperatively breeding cichlids (*Neolamprologus pulcher*) raised in socially deprived conditions, specifically with sibling brood mates only, show less submission after being attacked by dominants compared to fish raised in a social group with parents, subordinates, and same-clutch siblings [248], and down-regulation of their Glucocorticoid Receptor 1 gene in the telencephalon compared to fish raised in a social group with parents, subordinates, and siblings from the same clutch [249].

Several studies have also demonstrated both the short- and long-term effects that early social experiences have on the physiological response to stress [250,251,252]. Rodents exposed to adverse rearing environments exhibit heightened reactivity and impaired ability of the hypothalamic–pituitary–adrenal axis to effectively terminate the stress response [253,254]. Caldji and colleagues (2000) assessed stress in rats by measuring corticosterone levels in response to maternal separation [253]. Corticosterone serves as a sensitive and readily quantifiable indicator of maternal deprivation stress in rat pups, reflecting hypothalamic–pituitary–adrenal axis activity and responding to both deprivation and interventions mimicking maternal care [255]. Indeed, extensive animal research has demonstrated that exposure to insufficient maternal care or maternal deprivation early in life results in protracted stress responses [256]. This phenomenon is observed across species; for example, in rhesus monkeys and Goeldi’s monkeys (*Callimico goeldii*), both Suomi (1999) and Dettling and colleagues (1998) measured stress by assessing cortisol, the primate equivalent of rodent corticosterone, levels in response to social separation and maternal separation, respectively [252,256].

Furthermore, early maternal deprivation affects other neurochemical pathways in both animals and humans [257]. An example is the dopaminergic system, a complex network of neurons involved in a variety of functions, including reward and motivation [258,259], movement and coordination [260,261], learning and memory [262,263], attention and focus [264], and emotion [265,266], where deprivation can cause a reduction in dopamine receptor binding in certain brain regions. The effects of isolation impair animals’ cognitive abilities. These include (i) selective attention, which allows individuals to focus their attention on a particular task, object, or stimulus while ignoring other irrelevant or distracting information in the environment [267]; (ii) inhibitory control, which involves the ability to suppress or inhibit automatic or prepotent responses, thoughts, or behaviors to achieve a specific goal or task [232]; (iii) planning, which is the cognitive process of anticipating and organizing actions and behaviors to achieve a specific goal or objective; (iv) working memory, that is responsible for temporarily holding and manipulating information for the purpose of completing complex cognitive tasks; (v) problem solving, which is the cognitive process of identifying, analyzing, and resolving problems or obstacles that stand in the way of achieving a particular goal or objective [225].

The effects of early social deprivation have been demonstrated in numerous taxa such as fish [268], birds [269,270], rodents [271], primates [272], and humans [224] and the range of effects can extend from neuro-development, gene expression, physiology, cognition and even fitness. For example, mother-reared and peer-reared rhesus macaques acquired a higher social rank than young raised with limited access to conspecifics, suggesting that early social environment can have long-term effects that can affect even fitness [272].

While we have discussed the effects of social deprivation on cognitive abilities across several species, it is crucial to acknowledge the limitations in how these effects have been tested and reported in the literature [184].

Firstly, the developmental stage at which social deprivation occurs is a critical factor that can significantly influence outcomes. Different species have different sensitive periods for social development, and the timing of deprivation can lead to varied effects. For instance, in rodents, the early postnatal period is particularly crucial for social development [271], while in primates, social experiences during juvenile stages can have long-lasting effects [272]. However, not all studies specify the exact developmental stage at which deprivation occurred, making it difficult to compare results across studies or species.

Secondly, social deprivation rarely occurs in isolation. On the contrary, it is often accompanied by changes in other environmental factors such as reduced sensory stimulation, altered physical activity levels, or differences in nutrition. Differences in environmental complexity could contribute to cognitive differences independently of the social factor.

Thirdly, the duration of social deprivation and the specific nature of deprivation (e.g., complete isolation vs. reduced social contact) can vary widely between studies.

In conclusion, while there is growing evidence suggesting that early social experiences could play a role in shaping numerical cognition, more research is needed to fully understand this relationship. Future studies should: (1) manipulate early social experiences and measure their effects on specific numerical tasks across different species; (2) investigate how social deprivation affects the development of brain regions involved in numerical processing; (3) explore the interaction between social experiences and other factors (e.g., maturation, environmental complexity) in shaping numerical abilities; (4) examine whether the effects of early social experiences on numerical cognition persist into adulthood and how they might be mitigated.

## 5. Conclusions & Future Directions

This review highlights the crucial role of numerical abilities for a wide range of adaptive social behaviors in animals. The capacity to rapidly and accurately estimate numerosity and track small sets, together with skills such as proto-numerical discrimination, ordinal processing, proportional reasoning, and basic arithmetic, directly contribute to social behaviors. Individuals with more refined numerical competences are likely to interact more effectively in social groups, which in turn can increase their fitness and promote the stability of group structures. As a result, numerical abilities may become widespread within populations where group living confers survival advantages, thus creating a positive evolutionary feedback loop between numerical cognition and sociality.

While our review suggests that numerical cognition may be refined in response to the demands of living in complex social environments, this hypothesis requires further investigation and empirical support.

The Social Intelligence Hypothesis proposes that numerical cognition could be a core adaptive function for navigating social environments, rather than merely a by-product of ecological challenges [138]. However, this perspective needs more rigorous testing. Future research should directly compare the numerical abilities of closely related species with varying degrees of social complexity, controlling for ecological factors.

Research across various animal species has revealed some common mechanisms for numerical processing [12,39], which might suggest similar evolutionary pressures. However, we must be cautious in interpreting these similarities, as convergent evolution could also explain such commonalities. To better understand the evolutionary trajectory of numerical cognition, we need comparative studies that account for phylogenetic relationships and ecological factors.

A particularly relevant aspect concerns the distinction between early predispositions and acquired components of numerical cognition and the extent to which social interactions facilitate their development. Given the salience of numerical information in social contexts, numerical abilities are likely modulated by social factors during development. Furthermore, it can be postulated that the impairments observed in other cognitive domains due to social deprivation may also extend to numerical processing.

To advance our understanding of how social experiences shape numerical cognition, future research directions could be:

1. Longitudinal studies: These should track the development of numerical abilities from early life stages to adulthood in species with different social structures. For example, comparing the ontogeny of numerical skills in highly social primates like chimpanzees with less social ones like orangutans could provide valuable insights.
2. Integration of neuroimaging techniques: While challenging in non-human animals, techniques like fMRI or EEG could be used to study the neural correlates of numerical processing. For instance, comparing brain activation patterns during numerical tasks between socially reared and isolated individuals could reveal how social experience shapes neural circuits involved in numerical cognition.
3. Experimental manipulation of social experiences: Future studies could systematically vary the social experiences of young animals (within ethical bounds) and assess the impact on numerical abilities. This could involve comparing animals raised in standard social groups with those in enriched social environments or those with limited social contact.
4. Field studies: Observational studies in natural settings could provide ecological validity to lab-based findings. For example, tracking how wild animals use numerical information in social contexts (e.g., during intergroup conflicts or mating decisions) could offer insights into the adaptive value of numerical abilities.

A deeper understanding of how the social environment modulates the development of numerical cognition will have significant implications. It could provide optimal rearing and environmental practices to promote healthy cognitive development and shed light on neural mechanisms underlying the influence of early experience on the development of higher-order cognitive functions. In a broader context, it might also provide insights relevant to human cognitive development and inform educational practices and interventions for developmental disorders.

In conclusion, while the hypothesis that numerical cognition is a core adaptive function for social navigation is intriguing, it requires more robust empirical support. Future research should aim to test this hypothesis through carefully designed comparative and developmental studies. By doing so, we can gain a deeper understanding of the intricate relationship between sociality, cognition, and the evolutionary pressures that have shaped animal minds.

## Figures and Tables

**Table 1:** Social functions of numerical cognition across species. The table summarizes fundamental numerical abilities and their possible adaptive roles in social contexts across multiple taxa.

Social Function	Numerical Abilities	Species
**Assessment of group size during conflicts**	Proto-numerical Discrimination Proportional Reasoning	Lions (*Panthera leo*) [109] Spotted hyenas (*Crocuta crocuta*) [110] Chimpanzees (*Pan troglodytes*) [111] Black howler monkey (*Alouatta pigra*) [112] Subdesert mesite (*Monias benschi*) [113] Free-ranging dogs (*Canis lupus familiaris*) [114] Western Australian magpies (*Gymnorhina tibicen dorsalis*) [61]
**Protection from predators via group size**	Proto-numerical Discrimination	Guppies (*Poecilia reticulata*) [115,116] Sticklebacks (*Gasterosteus aculeatus*) [117] Red colobus (*Procolobus badius*) [118] Diana monkey (*Cercopithecus diana*) [118] Zebrafish (*Danio rerio*) [119]
**Navigation of dominance hierarchies**	Ordinal Processing	Baboons (*Papio anubis*) [106] Ravens (*Corvus corax*) [120] Greylag geese (*Anser anser*) [121] Domestic chickens (*Gallus gallus*) [122,123] Paper wasps (*Polistes dominula* and *Polistes metricus*) [124]
**Initiation of collective actions**	Proto-numerical Discrimination Proportional Reasoning	Ants (*Myrmecina nipponica*) [125,126,127] Capuchin monkeys (*Cebus capucinus*) [128] Baboons (*Papio anubis*) [129] African wild dogs (*Lycaon pictus*) [130] Meerkats (*Suricata suricatta*) [131] Honeybees (*Apis mellifera*) [132]
**Balancing foraging trade-offs**	Proportional Reasoning	Mallards (*Anas platyrhynchos*) [86] Cichlids (*Astatotilapia burtoni*) [89]
**Tracking group members**	Proto-Arithmetic	Domestic chicks (*Gallus gallus*) [133,134]

**Table 2:** Main pressures of the evolution of numerical cognition. The evolutionary pressure and the advantages of developing numerical processing are related to individual behavior and social behavior.

	Pressure	Advantages of Numerical Processing
**Individual Behavior**	Resources availability	Discriminating food resources (feeding)
	Mating opportunities	Discriminating number of males and females (reproduction)
	Habitat complexity	Counting landmark as reference point (navigation)
	Parasitism	Reject parasitized eggs (parental care)
**Social Behavior**	Collective foraging and hunting	Optimal distribution during foraging and numerical assessments during hunting strategies (feeding)
	Territory defense	Use numerical assessment to establish advantages during intergroup conflict (aggression and defense)
	Predatory risk	Join larger groups for defense from predators (defense)
	Group organization	Ordering group member in hierarchy (navigate social system)
	Group cohesion	Initiate collective action (decision-making)

## Data Availability

Not applicable.

## Abbreviations

The following abbreviations are used in this manuscript:

OFS	Object File System
AMS	Analogue Magnitude System
EIH	Ecological Intelligence Hypothesis
SIH	Social Intelligence Hypothesis
